# Giant Cell Arteritis versus Takayasu Arteritis: An Update

**DOI:** 10.31138/mjr.31.2.174

**Published:** 2020-06-30

**Authors:** Pavlos Stamatis

**Affiliations:** Department of Clinical Sciences, Rheumatology, Lund University, Sweden

**Keywords:** Giant cell arteritis, Takayasu arteritis, epidemiology, pathogenesis, histopathology, angiography

## Abstract

Giant cell arteritis (GCA) and Takayasu Arteritis (TAK) are two systemic granulomatous vasculitides affecting medium- and large-sized arteries. Similarities in GCA and TAK regarding the clinical presentation, the systemic inflammatory response and the distribution of the arterial lesions, have triggered a debate over the last decade about whether GCA and TAK represent two different diseases, or are age-associated different clinical phenotypes of the same disease. On the other hand, there are differences regarding epidemiology, several clinical features (eg, polymyalgia rheumatica in GCA) and treatment. The aim of this review is to present the latest data regarding this question and to shed some light on the differences and similarities between GCA and TAK regarding epidemiology, genetics, pathogenesis, histopathology, clinical presentation, imaging and treatment. The existing data in literature support the opinion that GCA and TAK are different clinical entities.

## INTRODUCTION

Giant cell arteritis (GCA) and Takayasu Arteritis (TAK) are two systemic vasculitides with predominantly granulomatous infiltrates that affect the aorta and its main branches. GCA and TAK comprise the group of large-vessel vasculitides. Traditionally, they are considered two different clinical entities. GCA and TAK are described both in the American College of Rheumatology 1990 classification criteria (ACR 1990) and in the 2012 Chapel Hill Consensus Conference definitions (2012 CHCC) as two different diseases.^1–3^ In both sets of criteria, age is used as an important discriminator between GCA and TAK. However, both GCA and TAK share several common clinical, histopathological and imaging features. The last decade, there is an ongoing debate about whether GCA and TAK represent two different diseases, or the same disease.^4,5^ Furthermore, regarding patients with large vessel vasculitis aged between 40–50 years of age, it is not always clear whether the large vessel involvement is due to late onset TAK or early onset GCA. The purpose of this article is to summarize the differences and common features between GCA and TAK with respect to epidemiology, pathogenesis, histopathology, clinical features, imaging, and treatment.

## EPIDEMIOLOGY

GCA is the most common vasculitis affecting individuals aged ≥50 years.^6–8^ The disease is very rare in individuals younger than 50 years of age. The mean age of disease onset is around the age of 75 years, in particular in patients with predominantly cranial symptoms.^7,9^ However, patients with large vessel involvement are generally younger at the time of GCA diagnosis.^10,11^ The disease is more common in Scandinavian populations and in populations with Scandinavian ancestry.^6,7^ The incidence of the disease increases with increasing latitude, with higher incidence rates in North European countries and lower incidence rates in Mediterranean and Asiatic countries.^6,12–14^ The incidence rate of biopsy-proven GCA (per 100.000 individuals ≥50 years) is 14.1 in Southern Sweden, 5.8 in Northern Italy and 1.1 in Turkey.^6,12,14^ The ratio between females and males is almost 3:1 in Northern Europe, but significantly lower in Southern Europe and Asia.^6,7,14,15^ A recent meta-analysis has shown that patients with GCA do not have increased long-term mortality in comparison with the background population.^16^ However, a study from Southern Sweden has demonstrated increased mortality the first 2 years after the GCA diagnosis,^6^ and the aforementioned meta-analysis has also demonstrated an increased mortality in hospitalized patients.^16^ Additionally, GCA patients have an increased risk of death due to cardiovascular disease.^17^

The incidence rate of TAK is significantly lower in comparison with GCA. The incidence of the disease (overall population without age restriction per 1 million inhabitants) has been reported to be 1–2 in Japan, 2.2 in Kuwait, 1.1 in Turkey, 0.7 in Sweden, 0.4 in Denmark and 0.4–1 in Germany.^18–24^ The incidence of the disease peaks in the 15–30 years-old age group.^22,25–27^ The female:male ratio has been reported to be 5:1 in Japan, and a higher ratio has been reported in Southern Sweden with 13:0 ratio.^19,22,27^ In contrast to GCA, TAK is very common in individuals with Asiatic ancestry.^26^ Mortality is approximately 3 times higher in patients with TAK in comparison with age- and gender-matched controls.^28,29^ Caucasian race and smoking have been identified as risk factors associated with mortality.^28^

## GENETIC FACTORS

Carmona et al. investigated the presence of genetic similarities between GCA and TAK in a meta-analysis of large-scale genotyping data.^30^

### HLA-associations

Single nucleotide polymorphisms (SNPs) in genes in the Human Leucocyte Antigen (HLA) class II regions, and in particular in the region between HLA-DRA and HLADRB1, were associated with GCA.^31^ On the other hand, SNPs in genes located in the regions of HLA class I, between HLA-B and MHC class I polypeptide-related sequence A; MICA, were associated with TAK.^30^ Particularly, the HLA Bw52 gene has been associated with susceptibility for TAK not only in Japanese populations, but even in European and American populations.^25,32,33^

### Non-HLA associations

Regarding SNPs outside the HLA-region, only one SNP was statistically significant affecting both GCA and TAK. This SNP was located in the region which encodes interleukin 12B (IL-12B).^30^ The affected gene encodes the P40 subunit, the common subunit between IL-12 and IL-23.

Taken all together, with the exception of the association of the SNP outside the HLA-region, there is no significant genetic correlation between GCA and TAK. GCA is associated with genes located in the HLA II region, whereas TAK is associated with genes located in the HLA I region.

## PATHOGENESIS

The above-mentioned HLA-class II genetic associations and the presence of clonal T-cells in different arterial sites suggest that GCA is an immune mediated disease.^34–36^ In large and medium sized arteries with vasa vasorum (diameter ≥ 2000μm), reside in the adventitia media border vascular dendritic cells (vas DCs).^37^ These vas DCs in healthy individuals are tolerogenic, sparing the host of the devastating consequences of inflammation in the arterial tissue.^36,37^ Several studies have shown that these vas DCs are impaired in GCA.^36,38–40^ In predisposed for GCA individuals, impaired vas DCs (eg, with polymorphisms in their Toll-like receptors) may be activated by the presence of danger signals, gaining T stimulatory capacity.^41–43^ This activation causes the migration of these DCs in the media where DCs produce chemotactic factors, which, in turn, cause the migration and activation of T-cells and macrophages.^36,37^ The subsequent inflammatory cascade orchestrated, mainly, by Th1-cell mediated and Th17-cell mediated responses contribute to the granulomatous infiltrate seen in GCA.^37,44^ There are also emerging data regarding possible immunostromal interactions (between T-cells, vascular smooth cells and endothelial cell, eg, Notch-Notch ligand interactions) and immunoinhibitory signals such as PD1-PDL1 pathway.^37,45,46^

The pathogenesis of TAK is poorly understood. Similarly to GCA, there is an inflammatory cascade initiated by impaired DCs and orchestrated by Th-1 and Th-17 responses resulting in the granulomatous infiltrate.^47^ However, there are some differences between GCA and TAK. In TAK, a currently unknown stimulus causes the overexpression of heat shock protein 65 kDA (HSP-65) which causes, in turn, the expression of cell surface protein MICA on vascular cells.^48^ MICA functions as a ligand for the NKG2D receptor, a receptor which is usually expressed in γδ T-cells, CD8-αβ T-cells and NK-cells.^49^ The recognition of MICA by γδ T-cells and NK-cells results in the production of perforin with subsequent vascular inflammation and damage.^47^ The dysregulated immune response and the uncontrolled activation of repair mechanisms contribute to the vascular damage seen in TAK. A very interesting finding is that in patients with GCA who are treated with glucocorticoids (GCs), the level of circulating Th-17 cytokines is significantly reduced after the treatment, whereas the level of Th-1 cytokines is unaffected in patients with chronic disease.^38,45^ The same statement stands even for the cellular populations of Th17 and Th1 cells in specimens of temporal artery biopsies (TABs) from patients with GCA.^44^ On the contrary, in TAK, the level of Th1-related cytokines is reduced after the treatment, whereas the level of Th-17 cytokines is unaffected.^50^

## HISTOPATHOLOGY

The TABs of patients with GCA show lymphocytic and/or granulomatous inflammation. Granulomatous inflammation may be present in the majority of TABs of patients with GCA.^51^ Regarding the location of the inflammatory infiltrate, the most frequent pattern is the pattern of transmural inflammation (75%), with the inflammatory infiltrate crossing the external elastic lamina and extending to the media.^52^ Inflammation in the media is the classical hallmark of GCA,^53^ and the inflammatory bulk in GCA is typically located in the adventitia media border.^54^ The inflammatory infiltrate usually consists of mature lymphocytes and macrophages. The macrophages are present in all arterial layers and may create rings along the internal elastic laminae.^55^ Giant cells are usually located along the internal elastic laminae and are present in up to 75% of the positive biopsies.^51,53^ The absence of multinucleated giant cells does not preclude the diagnosis of GCA. The granulomas in GCA are not usually compact/well formed. Plasma cells, eosinophils and neutrophils may also be present in various frequencies.^51,52^ Neoangiogenesis and myofibroblastic proliferation of the intima are frequent findings.^52^

The histological features of TAK are often almost indistinguishable from GCA with lymphocytic infiltrates, with or without giant cells.^19^ Multinucleated giant cells with engulfed fragmented elastic fibres may be observed in tunica media.^19^ However, some subtle differences in TAK are the trend for more adventitial involvement and the compact/well-formed granulomas.^54^ Severe adventitial scarring occurs more commonly in TAK.^26^ In end-stage TAK, the aorta has macroscopically a lead-pipe-like appearance due to the extended fibrosis, calcifications and atherosclerosis.^19^

## CLINICAL FEATURES

GCA is a heterogenous disease with 3 distinct clinical phenotypes, which may overlap with each other.^53^ Patients with GCA may have the classical cranial GCA, large vessel GCA (LV GCA), isolated PMR, or an overlap between these 3 clinical phenotypes.^55^ Headache of acute or subacute onset is present in approximately 70% of the cases as presenting symptom.^56–58^ Constitutional symptoms may be present in approximately 50% of the cases,^57,59,60^ whereas fever of unknown origin may be the presenting symptom in 10% of cases.^61^ Polymyalgia rheumatica (PMR) is a cardinal symptom at the disease onset in 40% of the patients,^59,62–64^ whereas 16–21% of patients with previously diagnosed PMR are going to develop GCA during their disease course.^55,65,66^ PMR and headache are the most common symptoms when the disease flares.^53,56,67^ One third of the GCA patients may have cranial ischemic symptoms at the time of GCA diagnosis (scalp tenderness and jaw claudication); symptoms which have been associated with severe ischemic complications such as stroke and visual loss.^56,60,64,68^ Visual manifestations are present in approximately 20% of the patients.^69,70^ However, the incidence of permanent visual loss has been reported to be reduced during the last decade probably due to the better recognition of the disease by clinicians.^71,72^ Large vessel involvement may be present in 30%–83% of the patients at the onset of the disease, depending on the imaging modality which is used.^4,73,74^ Finally, cerebrovascular accidents (CVAs), namely stroke and TIA, may occur early during the course of the disease, affecting most commonly the vertebrobasilar system in up to 7% of patients.^60,75–78^ *Table 1* presents the most common presenting symptoms in GCA.

**Table 1.** Baseline symptoms in GCA, based on selected studies.

**Study**	**Cases**	**Headache**	**Jaw claudication**	**Scalp tenderness**	**Visual symptoms**	**Constitutional symptoms**	**Arm claudication**	**PMR**
**Smith**, 1983^67^	24	83%	25%	33%	33%	29%	NR	25%
**Gonzalez-Gay,** 2005^64^	240	84.6%	41%	34%	23%	61%	3%	40%
**Schmidt,** 2008^62^	176	64%	41%	NR	29%	NR	NR	43%
**Zenone,** 2013^60^	98	76%	35%	NR	15%**	46%	2%	31%
**Tuckwell,** 2017^56^	119	71.4%	32.8%	36.1%	5.1%**	NR	NR	43%
**Pucelj,** 2019^58^	169	71.6%	45%	NR	33.1%	69.2%	NR	14.2%

** Ischemia related vision loss, NR: not reported.

In TAK, there is often a pre-stenotic phase (inflammatory) where the only symptoms may be constitutional symptoms and elevated inflammatory markers.^79^ If the carotid arteries are affected, carotidynia may be present at this stage due to the underlying inflammatory process.^80^ The pre-stenotic phase is followed by the ischemic/pulseless phase where arterial lesions (mainly stenosis and aneurysms) cause the signs and symptoms of the disease depending on the arteries which are affected.^79,81^ Consequently, *cerebral ischemia* may be presented as dizziness, headache, vertigo and hemiplegia; *upper limp ischemia* may be presented as extremity claudication, absent/weak peripheral pulse, finger numbness, cold sensation and extremity pain; *pulmonary artery involvement* may be presented as dyspnoea and haemoptysis; *coronary artery involvement* may be presented as chest pressure, angina, palpitations, shortness of breath and arrythmia; *renal artery involvement* may be presented as hypertension^27,82^; *mesenteric artery involvement* may be presented as abdominal pain, diarrhoea and hemorrhage.^19,83^ Some patients with TAK may develop skin manifestations such as erythema nodosum and pyoderma gangrenosum.^19,83^ Eye manifestations may be present in TAK, but permanent visual loss is quite rare in TAK.^81^ The eye manifestations in TAK may be caused by hypertensive arteriopathy, treatment-related cataracts, hypoperfusion secondary to cerebral ischemia, and retinal microaneurysms.^19,81,83^ A recent meta-analysis showed that the pooled prevalence of CVAs in the TAK was 15.8%.^84^ Finally, the need of surgical interventions in TAK is higher in comparison with GCA,^83^ and there is a worse prognosis in patients with extended vascular involvement and complications.^82^ *Table 2* illustrates the most common presenting symptoms in TAK.

**Table 2.** Selected studies on Takayasu arteritis presenting symptoms.

**Study**	**Country**	**Patients (n)**	**Constitutional Symptoms^†^**	**Upper limbs^∞^**	Head and neck^*^	**Eyes**	**Hypertension**
**Wong,** 2018^107^	China	78	12%	18%	6%	1%	62%
**Watanabe,** 2015^108^	Japan	1372	41%	17.3%	22.6%	3.3%	4%
**Kermani,** 2015^10^	USA	125	33%	65%	45%	NR	39%
**Mohammad,** 2015^22^	Sweden	13	54%	46%	NR	8%	38%
**Furuta,** 2015^11^	UK	23	65%	74%	0%	4%	NR
**Park,** 2005^109^	Korea	108	64.8%	72.2	56.5%	NR	NR
**Hall,** 1985^110^	USA	32	44%	94%	NR	NR	41%

† At least one of the following symptoms: fever, asthenia, fatigue, weight loss.

∞ At least one of the following symptoms: pulselessness, vascular bruits, blood pressure difference, fatigue, coldness and numbness.

* At least one of the following symptoms: dizziness, vertigo, syncope, headache, carotidynia and masseter claudication. NR: Not reported

## IMAGING

Four recent studies have evaluated common features and differences between GCA and TAK regarding imaging. Furuta et al.^11^ compared the clinical and radiographic findings in 22 patients with GCA and 23 patients with TAK. GCA patients were more likely to have headache, higher inflammatory response, previously diagnosed PMR and they were more likely to have long (>10 cm) tapered lesions in subclavian and carotid arteries. On the other side, TAK patients were younger, had longer diagnostic delay, had lesions in subdiaphragmatic arteries more frequently, and were more likely to have short non-tapered lesions in the carotid and subclavian arteries. Kermani et al.^10^ compared the clinical and radiographic findings in 120 patients with LV GCA and 125 patients with TAK. Again, GCA patients had higher inflammatory markers at the baseline and TAK patients had longer diagnostic delay. Occlusive lesions in left subclavian artery were more likely to occur in TAK patients, whereas aneurysms in the thoracic aorta were more common in GCA patients. Stenotic/occlusive lesions in thoracic aorta were more common in patients with TAK. All the GCA patients in this study had radiographically proved large vessel involvement, but less than one third of these patients had clinically detectable upper extremity abnormalities. Although this study compares the clinical phenotype of GCA which resembles TAK the most, there are several clinical and radiographic differences between GCA and TAK. Gribbons et al.,^85^ using a large multicentre multinational sample of 1068 patients with GCA and TAK, identified 6 patterns of arterial involvement, 3 patterns in GCA and 3 patterns in TAK. GCA patients were more likely to present: 1) low burden of the disease in large arteries (cluster four), 2) diffuse disease with involvement of the aorta and aortic arch (cluster five), and 3) axillary and subclavian disease (cluster six). On the other hand, patients with TAK were more likely to present: 1) involvement of abdominal, renal and mesenteric arteries (cluster one), 2) bilateral involvement of subclavian and carotid artery (cluster two), and 3) isolated involvement of left subclavian artery with minimal involvement of other arteries. Michailidou et al.,^80^ in a prospective observational study, investigated the association of clinical symptoms with magnetic resonance angiography (MRA) and fluorodeoxyglucose-positron emission tomography (FDG-PET) pathology, and subsequently compared the results between MRA and FDG-PET. In GCA patients, the most common symptom was blurred vision (37%) and in TAK patient arm claudication (52%). Arm claudication, CVAs and carotidynia were more common in patients with TAK. Disease activity expressed as elevated FDG uptake in several arteries was higher in patients with GCA, whereas vascular damage expressed as structural changes in the MRA was higher in patients with TAK. The presence of carotidynia in patients with TAK was associated with carotid abnormalities in FDG-PET, reflecting the underlying inflammatory process. Of note, the absence of carotidynia does not preclude imaging abnormalities in the carotid arteries. None of the GCA patients reported carotidynia. Posterior headache in patients with GCA was associated with imaging abnormalities in vertebral arteries in both MRA and FDG-PET. *Table 3* presents demographic, clinical and radiographic features of GCA and TAK.

**Table 3.** Differential features between GCA and TAK. Based mainly on the studies of Grayson et al.^4^, Furuta et al.^11^, Kermani et al.^10^, Carmona et al.^30^ and Gribbons et al.^85^

**Demographic, clinical and radiographic features**	**GCA**	**TAK**
Young age at disease onset (≤40 years)	−	++
Asiatic ancestry	−	++
Association with genes in the HLA II region	++	−
Cranial symptoms	++	+
Constitutional symptoms	++	+
PMR	++	−
Aortic insufficiency murmur	−	++
Eye manifestations	++	−
Acute phase reactants	++	+
Sub-diaphragmatic arteries (mesenteric and renal)	−	++
Axillary arteries	++	+
Aortic wall thickening	++	+
Stenosis/occlusion	+	++
Long (≥10cm) tapered lesions	++	+
Response to TNF-α inhibitors	−	++
Surgery or endovascular intervention	−	+

++: very common,

+: common,

−: uncommon. GCA: Giant cell arteritis, TAK: Takayasu arteritis

## TREATMENT

GCs remain the mainstay of treatment in GCA. The recent EULAR recommendations for the management of large vessel vasculitides advocate that the tapering of the GCs dose should reach to a target of 15–20 mg/day the first 3 months, and to a target dose below 5 mg/day after 1 year.^86^ Of note, EULAR recommends the initiation of GC-tapering when remission is achieved.^86^ In relapsing patients, in patients with life-threatening or organ-threatening manifestations, in patients with high future risk for glucocorticoid-related adverse events, and in patients where the prolonged use of GCs is expected to worsen pre-existing comorbidities, addition of a non-glucocorticoid immunosuppressive agent is recommended, including tocilizumab.^86,87^ The aim of the addition of a non-glucocorticoid agent is not only to reduce the disease activity but also to reduce the cumulative dose of glucocorticoids. Regarding synthetic disease-modifying anti-rheumatic drugs (sDMARDs), a meta-analysis of 3 randomized controlled trials^88–90^ has shown that addition of methotrexate is effective in patients with GCA reducing the risk for flares and having at the same time a significant GC-sparing effect.^91^ In an observational study, leflunomide appears to be effective in the treatment of GCA as a GC-sparing agent.^92^ Regarding biologic DMARDs, GIACTA trial has proved that weekly treatment with tocilizumab in combination with GCs reduces disease activity and has also a significant GC-sparing effect in comparison with treatment only with steroids.^93^ Three randomized controlled trials on TNF-α inhibitors (infliximab, etanercept and adalimumab) have failed to show any effect and, thus, treatment with TNF-α inhibitors is not recommended in GCA.^94–96^ There are ongoing phase 3 trials on abatacept (NCT02915159) and on the JAK-inhibitor upadacitinib (NCT03725202). With great interest, the METOGiA trial from the French vasculitis group is also much anticipated, where a head to head comparison of methotrexate and tocilizumab is planned (NTC03892785).

GCs remain also the mainstay of treatment in TAK. However, in TAK, the addition of a non-glucocorticoid agent such as methotrexate, azathioprine, leflunomide or mycophenolate mofetil is recommended at the time of diagnosis due to the high rate of relapse when patients receive monotherapy with GGs.^86,97–100^ In relapsing and difficult to treat cases, addition of a TNF-α inhibitor or tocilizumab should be preferred.^86,99^ An open-label trial^101^ and several retrospective studies have demonstrated the efficacy of TNF-α inhibitors in TAK.^102–104^ A recent randomized controlled trial of tocilizumab vs placebo failed to meet its primary outcome in the intention to treat analysis,^105^ which was the time to relapse. However, the relapse-free survival was 51% in the tocilizumab group vs 23% in the placebo group, suggesting a favourable effect of tocilizumab. The recently presented, in the annual ACR meeting in Atlanta, ACR 2019 guidelines for the treatment of TAK recommended the use of TNF-α inhibitors as first line biologics in TAK, reserving tocilizumab for refractory to TNF-α cases (unpublished data). In a randomized double-blind trial of abatacept, the risk of relapse was not reduced, when abatacept was added in the treatment with GCs; thus, Abatacept is not recommended in patients with TAK.^106^ The rate of vascular interventions is higher in TAK in comparison with GCA.^83^ If possible, vascular interventions should be planned when disease activity is low.

## CONCLUSION

Although inflammation of the aorta and its main branches is a common characteristic in both TAK and GCA, the existing data in literature support the opinion that TAK and GCA are two different diseases. There are striking differences in epidemiology (age, race), genetics (HLA II vs HLA I), histopathology (immune-cells comprising the infiltrate), clinical presentation (cranial symptoms and PMR in GCA), imaging (type of lesions and subdiaphragmatic involvement in TAK) and treatment (different responses in TNF-α inhibition).

## ABBREVIATIONS

ACR: American College of Rheumatology
AION: Anterior optic ischemic neuropathy
bDMARDs: Biologic disease-modifying anti-rheumatic drugs
CHCC: Chapel Hill consensus conference
CVA: Cerebrovascular accident
FDG-PET: Fluorodeoxyglucose-positron emission tomography
GCA: Giant cell arteritis
GCs: Glucocorticoids
HLA: Human leucocyte antigen
HSP-65: Heat shock protein 65 kDA
IL-17: Interleukin-17
IL-23: Interleukin-23
JAK-inhibitors: Janus kinase inhibitors
MICA: MHC class I polypeptide-related sequence A
MRA: Magnetic resonance angiography
NK cells: Natural killer cells
PMR: Polymyalgia rheumatica
sDMARDs: Synthetic disease-modifying anti-rheumatic drugs
SNP: Single nucleotide polymorphism
TAB: Temporal artery biopsy
TAK: Takayasu arteritis
Th1 cells: T helper type 1 cells
Th17 cells: T helper type 17 cells
TNF-α: Tumor necrosis factor alpha
Vas DCs: Vascular dendritic cells
